# Myxofibrosarcoma with associated Mycobacterium infection

**Published:** 2014-06-07

**Authors:** Sean Chen, Peter Mattei, Jens U Berli, Jaimie Shores

**Affiliations:** ^a^Department of Dermatology, Johns Hopkins School of Medicine, Baltimore, MD; ^b^Department of Plastic and Reconstructive Medicine, Johns Hopkins School of Medicine, Baltimore, MD

**Keywords:** myxofibrosarcoma, sarcoma, acid-fast bacilli, nontuberculous mycobacterial infections, skin and soft tissue infections

**Figure:**
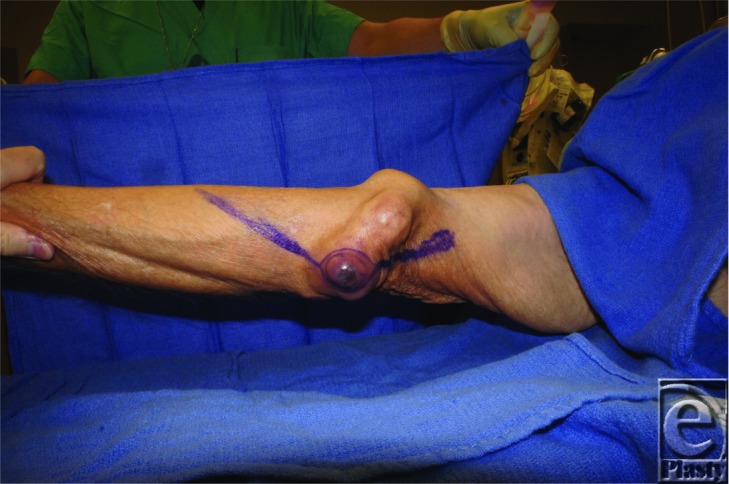


**Figure:**
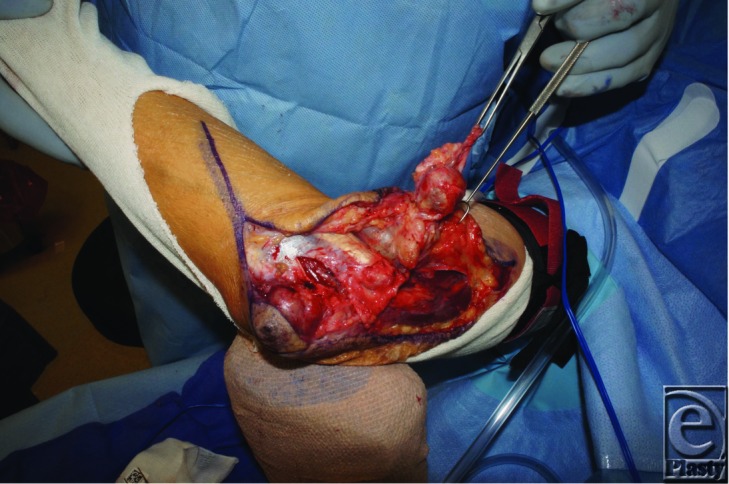


**Figure:**
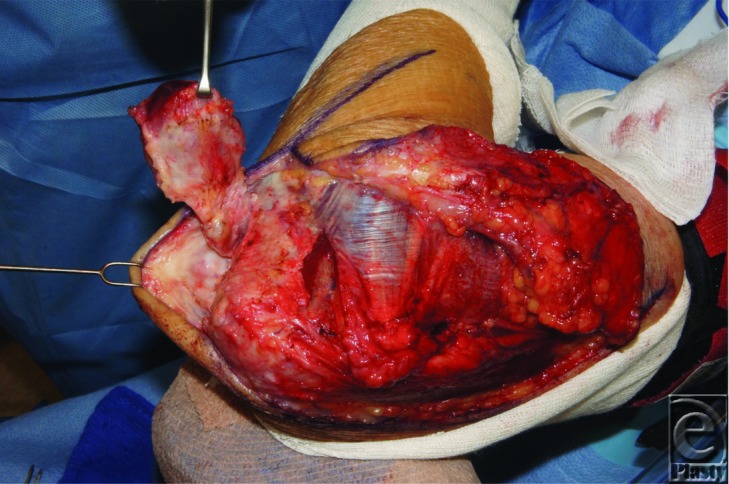


**Figure:**
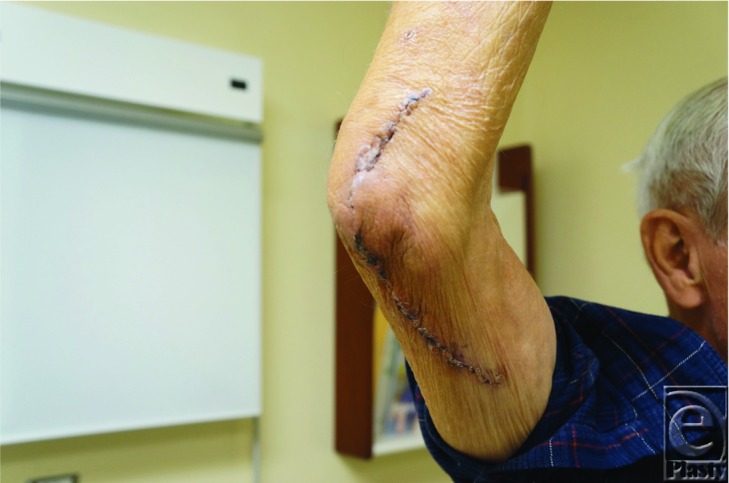


## DESCRIPTION

An 82-year-old male presented with a two-month history of a tender cystic elbow lesion. He reportedly drained it at home with a needle. MRI was consistent with a large ganglion. The mass was excised and pathology revealed a high-grade myxofibrosarcoma and intraoperative smears were positive for acid-fast bacilli.

## QUESTIONS

1. **Where do myxofibrosarcomas typically present?**
2. **What is the prognosis for myxofibrosarcomas?**
3. **What are the risk factors for acquiring cutaneous nontuberculous mycobacterial infections?**
4. **What is the presentation of cutaneous nontuberculous mycobacterial infections?**

## DISCUSSION

Myxofibrosarcomas are multilobular tumors of fibroblastic origin common in the elderly, and unlike other sarcomas, are often superficial lesions found in the subcutaneous tissue. Myxofibrosarcomas have historically been thought of as the myxoid variant of malignant fibrous histiocytoma. However, the nomenclature has been inconsistent. In 2002, the World Health Organization (WHO) defined myxofibrosarcomas as a discrete entity. Characteristic features include prominent curvilinear small capillaries with perivascular aggregates of tumor cells in a myxoid matrix. Clinically, they present as slow-growing, painless, palpable, and ill-defined subcutaneous nodules. Myxofibrosarcomas present in the extremities (77%), trunk (12%), retroperitoneum or mediastinum (8%) and head (3%).^1^

Myxofibrosarcomas display highly infiltrative growth and spread along fascial planes, possibly contributing to frequent rates of recurrence of up to 54%.^2^ When recurrent, the tumors may also evolve into higher grade lesions with more complex karyotypes.^3^ In a study of 69 patients, close or positive margins (<1mm) predicted worse local recurrence (HR 4.24, P = 0.030).^4^ In one study from Sweden where 10% of the 109 myxofibrosarcoma patients received adjuvant radiation after primary treatment had a local recurrence rate of 52% with median follow-up time of >5 years.^5^ Compared with a 5-year local recurrence rate of 14.6%, in a study where 80% of myxofibrosarcoma patients received adjuvant radiation.^6^ Thus patients with close surgical margins should be considered for radiation therapy. Metastasis is rare and occurs almost exclusively in higher-grade tumors. Mentzel et. al. reported 6 metastatic cases of myxofibrosarcoma to the lung, 5 to the lymph nodes, 2 to the skin and soft tissue, and 1 case to the bone.^2^ Similarly, Huang et. al. reported 6 metastatic cases to the lungs, 2 to the pleura, 1 to the pelvic bone, and 1 to the axillary lymph nodes.^7^

Nontuberculous mycobacteria (NTM) are acid-fast organisms that are ubiquitous in water, soil, food, and animals. NTM are classified as either rapidly growing or slow-growing. Rapidly growing species grow within several days and include *Mycobacterium fortuitum, M. abscessus, and M. chelonae*. In contrast, slow growing species take several weeks to grow and include *M. marinum and M. ulcerans*. Nontuberculous mycobacterial skin and soft tissue infections commonly occur in healthy individuals after cutaneous trauma in swimming pools, aquariums, or lakes and streams or through injections, surgical incisions, traumas, or puncture wounds.^8^

The incubation period for cutaneous infections by rapidly growing mycobacteria is approximately one month. Infections may present as ulcerations, plaques, folliculitis, papules, or nodules but can differ from other bacterial infections as they may be less painful and lack signs of systemic inflammation or lymphadenopathy.^8^ The diagnosis of nontuberculous mycobacterial skin and soft tissue infections requires isolation of the species in culture. Presence of AFB on smear even in the absence of growth on a culture medium is considered a clinically relevant infection. Clinical suspicion should be high if there has been a recent history of water exposure, penetrating injury or surgery, negative routine bacterial cultures, and lack of response to antistaphylococcal or antistreptococcal antibiotics. Without sensitivity data, it is recommended to treat with a macrolide antibiotic such as clarithromycin plus either fluoroquinolone, doxycycline or trimethoprim-sulfamethoxazole for 3-6 months depending on the extent of infection.^9^

Myxofibrosarcomas are fibroblastic tumors that typically present as subcutaneous nodules in the extremities of the elderly. These tumors display infiltrative growth and have a high rate of recurrence. Thus patients with close or positive surgical margins should be considered for radiation therapy. Metastasis, however, is rare occurring typically in higher-grade tumors. Cutaneous nontuberculous mycobacterial infections affect immunocompetent and immunocompromised individuals typically following exposure to water or through injury to the skin. A positive smear or culture should be treated with a macrolide with a fluoroquinolone, doxycycline or trimethoprim-sulfamethoxazole for a prolonged course of 3-6 months.
